# Falsification and experimental psychology: examples from face perception

**DOI:** 10.1007/s12124-026-10024-1

**Published:** 2026-09-25

**Authors:** Sam S. Rakover

**Affiliations:** Department of Psychology, Haifa University, Haifa, 3498838 Israel

**Keywords:** Confirmation, Falsification, Methodology, Duhem problem, Contradictory findings

## Abstract

The present article examines the relationships between the falsification approach as outlined in Popper (1995) and the methodological basis of experimental psychology. In view of this, I will offer suggestions that may reduce somewhat the severity of the following two problems that were raised against Popper approach: The “Duhem problem”, and “contradictory-findings disregard”, i.e., the fact that scientists tend to ignore findings that disconfirm their theory. These suggestions are based on the development of the “temporary closed explanatory domain” (TCED) model, which describes how the research in experimental psychology is carried out. Accordingly, The TCED proposes that a theory T (model, hypothesis) outlines a temporary closed domain for explaining the behavior under study. Two important factors outline the boundaries of TCED in experimental psychology. The first factor includes the variables of the theory itself that are defined operationally, and the experimental framework. The second factor refers to the “basic experimental manipulation” (BEM) that generate the behavior under study, the behavioral phenomenon that T attempts to explain. The article analyzes the Catch model for recognition and reconstruction of faces, as a research in experimental psychology that illustrates well the TCED approach.

## Introduction: main ideas and some clarifications

The purpose of the present article is to examine the relationships between the falsification approach as outlined in Popper (1995) and the methodological basis of experimental psychology. Considering this and the way experiments are conducted in psychology, I will offer suggestions that may reduce somewhat the severity of the following two problems that were raised against Popper approach: The Duhem problem, and the fact that scientists tend to ignore contradictory findings.

The article will be organized as follows. First, I will describe the experimental methodology in psychology, then the Popperian approach, and the current approach: the “temporary closed explanatory domain” (TCED) model. Second, a special section will be devoted to the critique of falsifiability and the use of the null hypothesis in psychology. Here I will describe how TCED addresses the Duhem problem and the phenomenon whereby researchers ignore the observations that contradict their theory. Third, I will describe the “Catch” model of face recognition and reconstruction as an example that illustrates TCED. Finally, the article will conclude with a discussion of the current approach.

Since the methodology of psychology is based on the methodology that developed in the sciences, part of the discussion will be based on the arguments that appear in the literature of the philosophy of science (e.g., Rakover, 1990, 2018). I will focus in particular on Popper’s (1995) approach, on the basis of which I will develop the present proposal, the “temporary closed explanatory domain” (TCED) model, which describes/explains how the research in experimental psychology is carried out (e.g., Rakover, 2003). First, I will present briefly what I believe to be the accepted research methodology in psychology and thereafter I will present the TCED. Here I should note that at this point I would not enter into a detail discussion of the debate about whether there is indeed an accepted methodology in science in general and in psychology in particular. I will only mention that from my personal experience, including conversations with colleagues, it seems to me that the following description characterizes the methodology taught in undergraduate psychology.

### Psychology accepted methodology

The accepted view of science is founded on rational scientific methodology (e.g., Hempel, 1979; Hepburn & Andersen, 2021; Siegel, 1985). The problem that arises here is whether there is a uniform, canonical methodology of science. Several researchers suggested that there is no uniformity in scientific methods (e.g., Feyerabend, 1975; Hepburn & Andersen, 2021; Siegel, 1985).

Nevertheless, in my opinion, there is a general research scheme that most researchers use it in one way or another in psychological research. This scheme can be realized in different forms depending on the field of research itself. For example, the research practice in face perception is different from that used in infant development, although both cases can be seen as different realizations of the same general research scheme. In view of this approach, as an experimental psychologist, I will now describe a general experimental method with which I have conducted many studies on avoidance learning in rats and human memory and perception. Briefly, this methodological approach is based on the conventional research methods studied in many departments of psychology (e.g., McCall & Kagan, 1990; Neal & Liebert, 1986; Rakover, 1990) and it is consistent with the most general conception of the scientific method (see Hepburn & Andersen, 2021, subsection 6.1).

This methodological approach is based on the well-known Hypothetico-Deductive (HD) method and the Statistical Hypothesis Testing (SHT), which I call for short the “HD&SHT” method (e.g., Hepburn & Andersen, 2021; Rakover, 1990). In short, according to the HD&SHT method, a researcher deduces from a theory (hypothesis, model) under study and certain empirical conditions a specific prediction that he/she tests empirically by means of a well-designed and controlled experiment. (This particular prediction may arise for several reasons, such as, a new and interesting observation, and an attempt to decide between two competing theories in a particular empirical field.) If the prediction is consistent with the empirical result, then it can be suggested that the theory has been confirmed; however, if the prediction does not fit the result, the theory is disconfirmed. The decision whether or not there is a match between the prediction and the experimental result is determined by the appropriate statistical tests. According to the conventional method (HD&SHT), the researcher performs a statistical test and decides as follows. If H_0_ (the null hypothesis) is rejected (and H_1_, the tested, alternative hypothesis is accepted) the researcher interprets this statistical finding as confirmation, as support for the tested hypothesis; and vice versa, if H_1_ is rejected, the researcher interprets this finding as a rejection of the tested hypothesis. As a simple example, let us examine the following.

The holistic/configural theory of face recognition suggests that inverting a face (hair down, chin up) impairs the holistic perception of the face and its configural information (the spatial relationships between the facial features). The immediate and clear prediction that emerges from this theory is as follows: recognizing an upright face (U_F_) is better than recognizing an inverted face (I_F_). The results confirmed this prediction: an U_F_-average recognition is statistically significantly greater than I_F_-average recognition (e.g., Maurer et al., 2002; Rakover, 2002; Rakover & Cahlon, 2001; Yin, 1969).

Given the accepted scientific methodology (especially the HD method) one may propose that it is closely related to Popper(1995) scientific approach of falsifiability (e.g., Fiedler et al., 2012; Leppink et al., 2017; McGuire, 2013; Wason, 1960; Wilkinson, 2013). For example, McGuire (2013, p. 414) wrote about the relationship between philosophy of science and psychology (a relationship that he criticized), “… the reigning philosophy of science in psychology, which is most strongly associated with Karl Popper. … the ubiquitous assumption that the purpose of empirical research is to determine which hypothesis and theories should be rejected (or falsified) and which should be left standing (for the time being, at least).” In view of this, I will now describe briefly Popper’s falsification approach.

### Falsification

I believe that Popper’s (1995, pp. 32–33, 53) central ideas are reflected in his description of how scientific research has to be conducted. “From a new idea, put up tentatively, and not yet justified in any way – an anticipation, a hypothesis, a theoretical system, or what you will – conclusions are drawn by means of logical deduction. These conclusions are then compared with one another and with other relevant statements, so as to find what logical relations (such as equivalence, derivability, compatibility, or incompatibility) exist between them.” (p. 32). And he continued to write about the decision regarding the comparison between theoretical conclusions and empirical findings, “If this decision is positive, that is, if the singular conclusions turn out to be acceptable, or *verified*, then the theory has, for the time being, passed its test: we have found no reason to discard it. But if the decision is negative, or in other words, if the conclusions have been *falsified*, then their falsification also falsifies the theory from which they were logically deduced.”

“It should be noticed that a positive decision can only temporarily support the theory, for subsequent negative decisions may always overthrow it. So long as a theory withstands detailed and severe tests and is not superseded by another theory in the course of scientific progress, we may say that it has “proved its mettle’ or it is *corroborated* by past experience.” … “The game of science is, in principle, without end. He who decides one day that scientific statements do not call for any further test, and that they can be regarded as finally verified, retires from the game.” (p. 53).

Here and in other places in his book, Popper (1995) makes the distinction between a scientific theory, which in principle has to withstand the empirical possibility of its falsification, and a non-scientific theory that has not. Falsifiability, then, delineates the empirical domain from which observations that disconfirm the scientific theory can arise. A scientific theory has to propose a specific prediction that under certain conditions can be falsified. If a theory cannot do that, it is not a scientific theory. When a theory withstands falsification, it does not mean that the theory has been verified, but rather that it is temporarily corroborated (until the moment when it is falsified).

Popper’s approach is based on well-known rules of logic. There is no logical rule that says: if T entails P, and P *did occur*, then T is true. However, there is a logical rule (the Modus Tollens) that says: if T entails P, and P *did not occur*, then T is false. That is, if a certain prediction P derived from T does not occur in an experiment (or by observation), then T is disconfirmd. Popper therefore suggested that scientists do not seek to confirm their theory experimentally, but rather they attempt to put the theory in the test of falsification. If the prediction is not occurred in reality, then logically the theory is disconfirmd, but if the prediction did occur, the theory should be held not as a true theory, but as one that has stood the falsification test temporarily, until the observation that will disconfirm it is discovered.

### TCED

The TCED (temporary closed explanatory domain) model proposes that a theory T (model, hypothesis) under study outlines a temporary closed domain of empirical observations, of behaviors that T successfully explains. It is a temporarily closed domain, because ongoing research may lead to theoretical and empirical changes of its boundaries. Two important factors outline the boundaries of the closed explanatory domain in experimental psychology. The first factor includes the variables of the theory itself that are defined operationally and the experimental framework. The second factor refers to the “basic experimental manipulation” (BEM) that generates the behavior under study, the behavioral phenomenon that T attempts to explain. For example, the BEM in the ongoing research over decades of the ‘face inversion effect’ (FIE) is the presentation of an inverted face (e.g., Maurer et al., 2002; Rakover, 2002, 2018; Yin, 1969). In other words, T draws a closed theoretical-observational framework, within which the empirical research can be executed and the behavioral phenomena be explained.

As described above, the TCED model is anchored to several points in Popper’s approach (falsification), nevertheless, it offers also some new directions.

First, Popper’s falsifiability approach can be interpreted as recommending that the goal of research is not to confirm a theory, but rather to disconfirm it. However, this conclusion does not follow directly from the TCED model. The purpose of TCED is to explain the phenomena being studied in the temporarily closed research area. This purpose is realized is by using HD&SHT, the method that describes the conventional way by which research in psychology is done. In this respect, the question of whether the researcher is interested in confirming or disconfirming a theory moves from the level of logic/methodology to the psychological level, i.e., it becomes largely a psychological question. If the theory is the researcher’s favorite, he/she is happy to confirm it. However, if the theory is a rival theory, the researcher is happy to disconfirm it.

Second, in accordance with Popper’s falsification approach, TCED also does not accept that a successful theory, no matter how successful, is a true theory. A successful theory is provisional, because one cannot ignore the possibility that a specific phenomenon may be discovered in the future that will disconfirm the successful theory.

Third, in accordance with Popper’s falsification approach, the domain, from which possible potential observations that confirm/disconfirm the theory may arise, is determined by the theory itself. However, the main difference between Popper’s approach and TCED is as follows: TCED emphasizes the centrality of BEM (basic experimental manipulation) in the closed empirical domain of a psychological theory as a crucial factor in the process of explaining the behavior under study.

Given these introductory comments, I will now discuss two important criticisms of Popper’s approach and the problem of H_0_ in SHT.

## Criticisms on falsification and the null hypothesis

To focus the review on the issue of falsifiability and the null hypothesis, which are cornerstones of the accepted methodology, DH & SHT, I will examine the following simple example.

Suppose that a researcher proposes a hypothesis that manipulation M causes behavior B (i.e., M → B). To test this prediction empirically, he/she activated M in the experimental group and compares B to the corresponding control group in which M was not activated. Then, he/she tested statistically two hypotheses. According to H_1_: B in the experimental group (B_EX_) is greater than in the control group (B_CO_), i.e., B_EX_ > B_CO_. According to H_0_: there is no difference between B_EX_ and B_CO_, i.e., any difference between the two groups is the result of random processes. Given this, the following question arises: can one propose that if H_0_ is rejected (since the probability of the observation/H_0_ is *p* < .05, see Gigerenzer et al., 2004) then H_1_ is confirmed, and if H_0_ is accepted (since *p* > .05) then H_1_ is disconfirmed (falsified)?

I will first answer H_0_-problem on a purely logical basis, without referring to the second problem, which is the criticisms on falsification.

*First*, from H_0_ point of view, there are two possibilities.

(I) *The null hypothesis (H*_*0*_*) is correct*. Given that {[If the experimental hypothesis (H_1_) is correct, then H_0_ is incorrect] and it has been discovered that H_0_ is *correct*, i.e., *p* > .05} it follows that H_1_ is incorrect. Why? Because this conclusion is based on the logical principle, the Modus Tollens, according to which. If T → P and P *does not occur*, then T is *incorrect*.
(II) *The null hypothesis (H*_*0*_*) is incorrect*. Given that {[If H_1_ is correct, then H_0_ is incorrect] and H_0_ is *incorrect*, *p* < .05} it follows that H_1_ is not correct (true) but rather is confirmed, is temporarily corroborated (until the moment it is falsified). Why? Because the following logic is *incorrect*, If T → P and P does occur, then T is correct. For example, even if we examine many swans in Europe and reveal that they all are white, this finding does not validate the hypothesis, ‘all swans are white’, simply because black swans were revealed in Australia (this is an example of the induction problem).

In an article devoted to discussing the H_0_ problems, Gigerenzer et al. (2004) pointed out that psychologists do not take into account the heated debates regarding the use of H_0_, which includes different approaches of Fisher, Neyman and Pearson, Bayes, and the power of a statistical test. They continue to use ritually the statistical test of accepting/rejecting H_0_. In many respects this criticism is correct, but in recent years there has been some improvements. First, many articles discussed contradictory findings from methodological and theoretical perspectives (e.g., McGuire, 2013). Second, a large number of experimental articles reported in addition to the α-coefficient also the β²-coefficient, which is a measure of the variance explained by the predictor (independent) variables. This information expands the researcher’s knowledge beyond the ritualistic reporting of *p* < .05 for confirming H_1_.

**Fig. 1:**
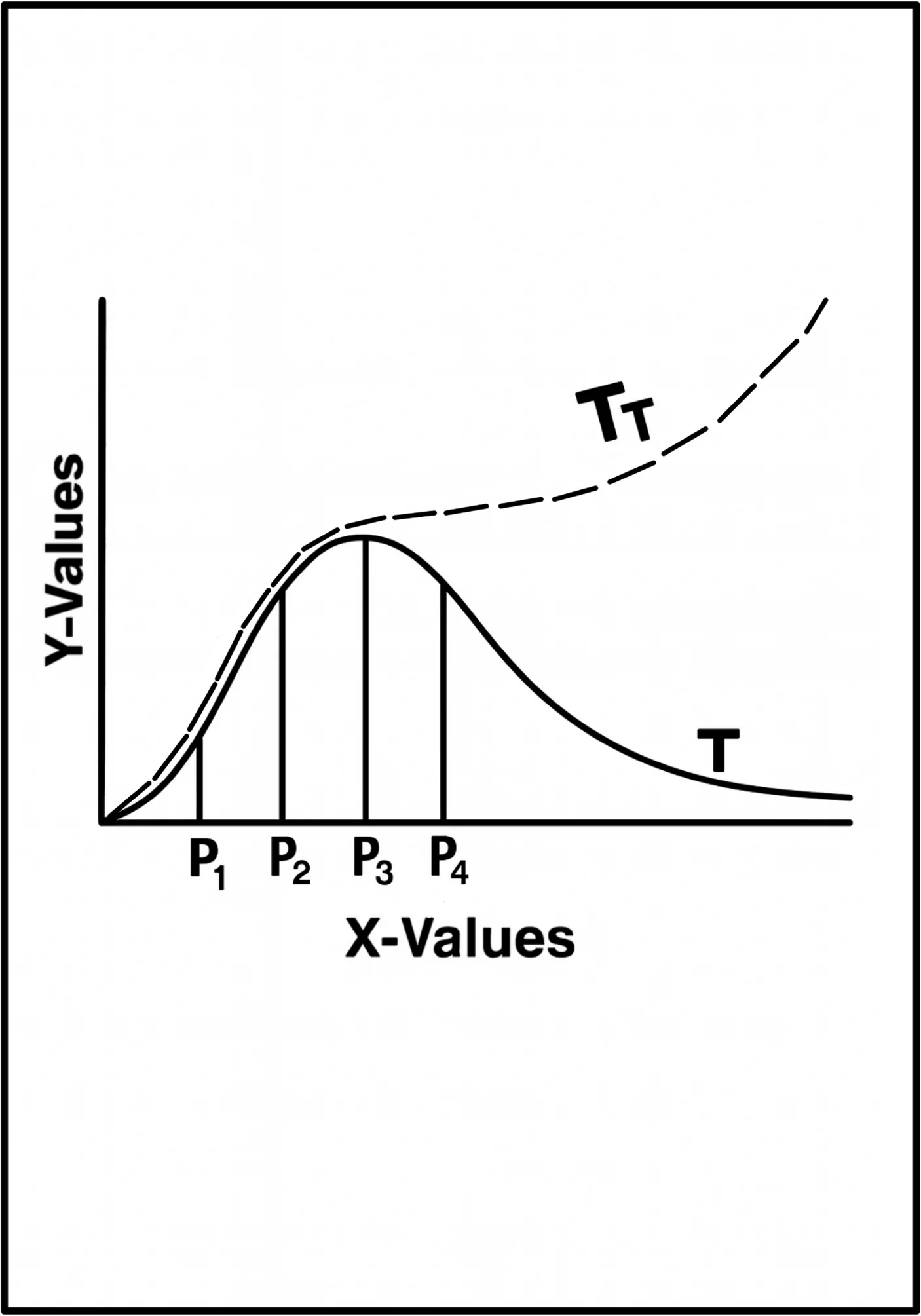
depicts Y-values as a function of the unknown true theory T_T_ and the proposed theory T that made four predictions, P_1_ to P_4_ (see the X- axis). TCED includes the four predictions
  The TCED is based on Rakover (2003) who attempted to suggest a practical solution to Duhem problem. The basic idea is that research is done in a closed theoretical and empirical domain. Therefore, it is possible to test a limited number of factors that may be responsible for the failure of the theory under test. Practically, each component of the experiment can be examined according to its nature. For example, the question of whether the problem lies in the construction of the computer software will be examined in a completely different way than the question of whether the participants in the experiment had a certain trait, a tendency that biased their responses. One may test the components of the entire theoretical system, such as the theory itself, the axillary hypotheses, and the experimental equipment, in a way similar to testing a malfunction in a car. Just as it is possible to test why a car as a whole does not start, it is possible to test why the experimental system produced results that contradict the theoretical prediction. In both cases, the tests are done in closed systems that allow for the examination of each of their components. As can be seen, this proposal for dealing with the Duhem problem is not based on pure logic but on workable methodology.
(II) *Contradictory-findings disregard (CFD)*. A number of philosophers of science discussed the phenomena that scientists have disregarded empirical findings that contradict the predictions derived from their theory (e.g., Lakatos, 2014). For example, even Newton developed his theory of gravity in an idealized way (which is very different from reality), with the masses of the Sun and planets represented as theoretical points. Lakatos (2014, p. 193, footnote, 116) presented Reichenbach’s interesting explanation for Newton’s delay in publishing the *Principia* by about twenty years. The reason was that Newton discovered that empirical observations did not match the predictions calculated on the basis of his gravitational theory.
  Lakatos himself explain the CFD phenomenon in the following way. He proposed that scientists protected the ‘hard core’ of the research programme from falsifying observations by adjusting the ‘protective belt’. Contradictory observations were interpreted in terms of certain axillary hypotheses in the protective belt, interpretations that transomed them into non-falsifiers, into supportive observations. Similarly, Popper (1995) allowed certain methodological non-ad hoc adjustments of auxiliary hypotheses, which produce new interesting predictions. Khun (1970) suggested that paradigm normal science resists contradicting observations (anomalies), until the moment that their accumulation generate a scientific revolution. By comparison, the TCED proposes somewhat a different perspective.
  The TCED’s explanation moves from the epistemological-methodological level to the psychological level, as it based on the attitude of psychologists towards their favorite theory (model, hypothesis) in the domain of its explanation. The TCED divides the behavioral space into two parts. One part is the unknown field that includes a huge number of behaviors and possible explanations of all kinds, an “unknown land” in which one can get lost. The second part, the TCED, is a limited area of investigation in which the researcher’s theory has managed to offer certain explanations to parts of the phenomena (see Fig. 1) and is also related in one way or another to other theoretical approaches that have received some degree of empirical support.
  As can be seen from Fig. 1, the question that arises is this. What will be the researcher’s attitude towards an empirical finding, P_4_, which contradicts his/her favorite theory, T? The simple falsification approach says that there is no choice, the theory must be abandoned because it is incorrect, it has been disconfirmed. However, this is not what researchers do, because they find themselves in the following difficult situation. On the one hand, if the researcher abandons his/her theory, then he/she returns to “unknown land”, and he/she must start over again. This is an unattractive and actually repulsive option. The researcher must admit failure and gather again the strength, ideas (which in the current situation do not appear in his head) and means (money, etc.) to start over. On the other hand, if the researcher remains in TCED, then he has a theory that manages to explain some of the phenomena, which is a lot. As for the contradictory findings, several rational actions can be taken. For example, one may examine how these falsifying findings were obtained and whether some appropriate changes in the theory (or the axillary hypotheses) can be made so that the theory can successfully handle the contradictory findings. There is no immediate need to throw up one’s hands and abandon the theory that has been developed so far with so much effort (and yes, this is consistent with Festinger’s, 1957 dissonance theory).
  Given this, it follows naturally from the researcher’s psychological considerations that the contradictory findings can be ignored and that the researcher will continue hold on to the theory, as long as the researcher intends to address these findings in one way or another within the framework of his theory.

*Second*, many criticisms have been published on the Popperian approach. However, since the aim of the present paper is to describe the way in which TCED deals with two important problems raised against falsifiability, I will concentrate on these two: the Duhem problem and ‘Ignoring contradictory findings.’

(I) *Duhem problem*. This is a methodological criticism that undermines the possibility of falsifying an isolated hypothesis by an observation that contradicts the hypothesis’ prediction. Duhem (1996, p. 81) wrote, “That an experiment in physics can never condemn an isolated hypothesis, but only a whole theoretical group” (see also Rakover, 2003). Accordingly, one tests the whole theoretical system: the target theory (model, hypothesis) together with auxiliary hypotheses and background theories, and therefore, it is not possible to decide, which of these is the reason why the prediction is not consistent with the observation. For example, the failure to make the correct prediction may be due to the following. Problems in the theory, in the experimental setup, in the software that presents the stimuli to the subject, in the calculations that were made on the basis of the participant’ responses, or in the assumption that the recruitment of participants was random, and so on.
  Popper’s (1995) handled Duhem problem by proposing that methodologically one falsifies the whole theoretical system, but by employing certain methodological and rational considerations, one may discover which part of the whole system is responsible for the unsuccessful prediction. In comparison, Lakatos (2014) suggested to differentiate in a ‘research program’ between the ‘hard core’ that includes the fundamental theoretical assumptions that are protected against negative observations, and the ‘protective belt’ that include auxiliary assumptions and hypotheses, which can be modified easily and by that protect the hard core. (Note that a ‘progressive program’ does not use ad-hock modifications, whereas ‘degenerative program’ uses them.) So, Lakatos located the place of Duhem problem in the protective belt, wherein appropriate changes could be made as part of an ongoing research program.
  The TCED leans on both these approaches. It emphasizes the importance of the boundaries of the research domain outlined by the theory and BEM under study. They minimize the number of the auxiliary hypotheses and experimental means to a manageable number, i.e., a number of factors that can be treated practically. For example, the researcher may evaluate each component of the whole system by referring to the appropriate literature, by conducting a systematic examination of each experimental means (e.g., the experimental software, the various calculation programs), or by conducting parallel checking experiments in which only one target variable is manipulated (in Fig. 1, the TCED is the area under the T function).

## The “Catch” model for face recognition: An illustration of TCED

The purpose of the current example is to illustrate the TCED including BEM, and in particular to describe what happened after the discovery that certain empirical observations contradicted the Catch model.

I will start with two preliminary points: First point: The Catch model is based on a process that is the opposite of the usual approach for explanation of behavior. While the standard procedure seeks to explain a Response as a function of a Stimulus and a Theory, i.e., R = f(T, S), the Catch model seeks to reconstruct the Stimulus (the suspect’s face) from the witness’s memory using a Theory (for a face reconstruction) and the witness’s Response, i.e., S = f(T, R).

Second point: The procedure of the Catch model is the opposite to the police standard procedure for face reconstruction. The standard technique reconstructs the suspect’s face from the eyewitness’s memory in two steps. The first-step is based on matching facial features, such as the eyes, noses, and mouths, to the suspect’s facial features stored in the eyewitness’s memory. For example, the eyewitness selects from several pictures of eyes the one that most closely resembles the eyes of the suspect’s face. The second-step is based on combining all these selected facial features into a single composite, which according to the eyewitness resembles the suspect’s face.

**Fig. 2:**
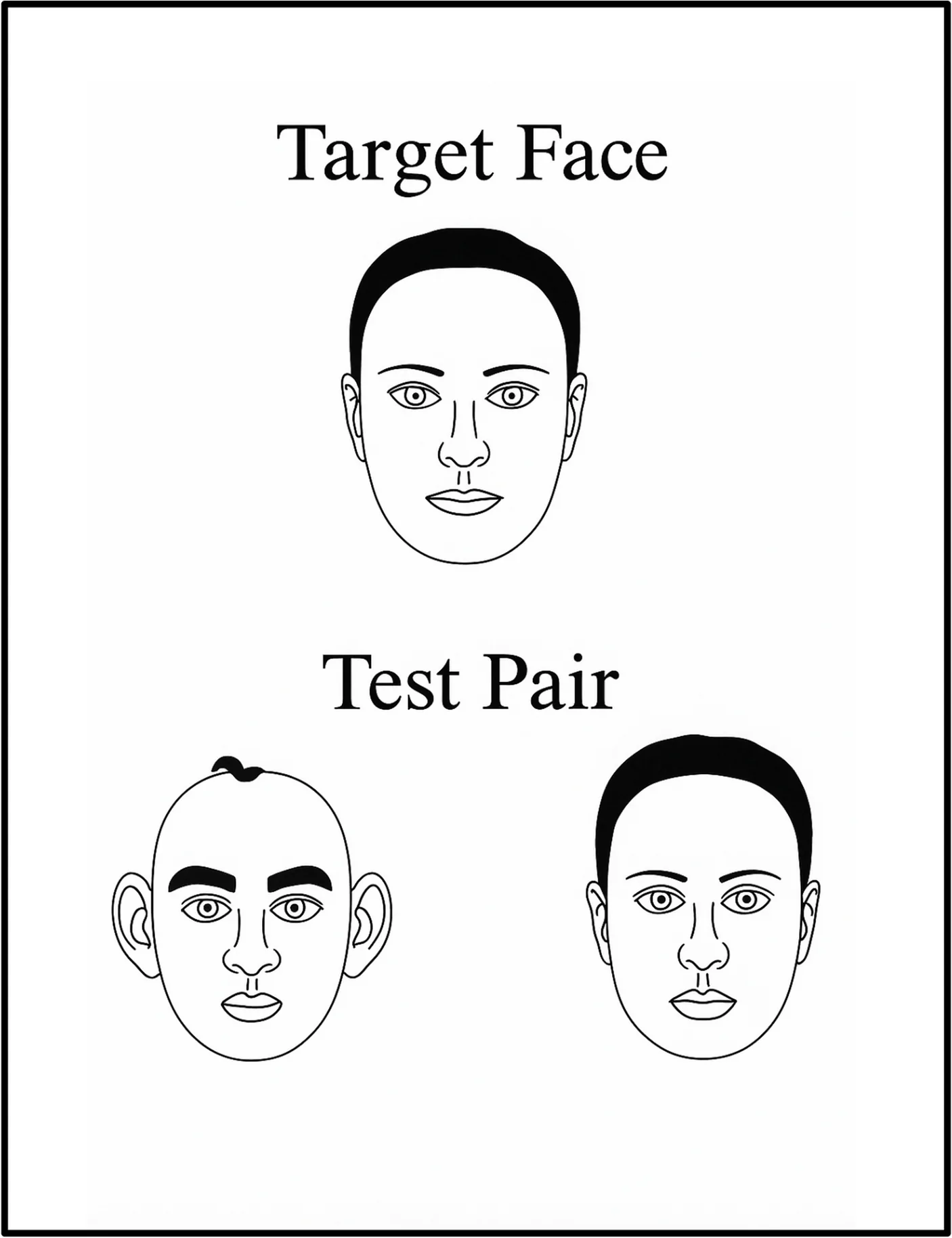
depicts schematically the target face (which is stored in memory) and a test pair. The example shows that the right face is more similar to the target face than the left face. While the right face and the target face share four facial features (hair and forehead, eyes, nose, and chin and ears), the left face shares only one facial feature (nose). The drawing is based on Rakover and Cahlon (2001, Fig. 6.1)

However, the procedure of the Catch model is the opposite. As can be seen from Fig. 2, the witness is presented with a test-pair of faces from which he/she has to choose the one that most resembles the suspect’s remembered face (the target face, F_t_). Then, based on a number of such selections, the suspect’s facial features are extracted and the reconstruction of his/her face is obtained (for details, see below). The idea underlying the proposed model is that humans perceive the face as a whole unit (e.g., Rakover, 2013; Rakover & Cahlon, 2001; Tanaka & Farah, 1993), and therefore it will be easier to reconstruct the suspect’s face by this procedure than by matching pictures of facial features to the suspect’s face stored in memory.

The main assumptions and procedures of the Catch model are as follows.

### A face (F) and the space of faces (S_F_)

A frontal face is divided into 5 dimensions (hair and forehead, eyes, nose, mouth, and chin) when each dimension has n different values (e.g., 70 different eye shapes). The “space of faces” (the number of all possible faces) is given combinatorically by, S_F_=n^5^. If, for example, each dimension has 3 values, then S_F_=3^5^=243 faces and one of them is the target face (F_t_). We defined a face F as a vector of 5 different values. The target face F_t_ is the suspect’s face stored in the eyewitness’s memory. This is the unknown face, which the Catch model attempts to reconstruct. For the sake of simplicity, F_t_ is defined by the vector F_t_: 11111.

### Decision by similarity rule

The eyewitness decides which of the two test-faces presented to him is more similar to F_t_ according to the following similarity rule: the selected face is the one that has more values in common with F_t_ (see Fig. 2). For example, if F_t_: 11111 (*n* = 3) and the test-pair is: on the left F_L_: 12311 and on the right F_R_: 31323, then F_L_ is selected since it has 3 common values with F_t_, whereas F_R_ has only 1. (Note that the Catch model has a few complications that I will not discuss here. They were resolved successfully; see Rakover & Cahlon, 1989, 1999, 2001.) According to this description, it is clear that the basic experimental manipulation, BEM, in the series of experiments that will be reported below is this: The participant has to choose from two test-faces the face that most resembles the defendant’s face stored in his/her memory.

### Reconstruction rule for F_t_

This rule has three stages. In the rule’s first stage, for each chosen test-face as more similar to F_t_, one determines the values that it and F_t_ share and that do not appear in the rejected test-face [called the “differentiating-values” (DVs)]. For example, in the above test-pair, the DVs are: 12–11, the (-) signifies a common value of both test-faces. In the rule’s second stage, one summarizes the frequency of occurrence of the DVs for each dimension. The value with the largest frequency is the value of F_t_. In the rule’s third stage, F_t_ reconstructed by using the 5 DVs that are the F_t_ values.

These three assumptions and procedures constitute the TCED of the present area of research in face reconstruction. It is a closed theoretical system, which delineated its empirical research domain. Rakover and Cahlon (1989, 1999, 2001) have proven mathematically that indeed the Catch model reconstructs F_t_! The question is, of course, whether the experimental procedure will lead to a complete identification of the F_t_; whether the empirical results will reflect the theoretical model. The answer is as follows.

To test empirically the Catch model, a series of experiments was conducted; each one is based on two stages (e.g., Rakover & Cahlon, 1989, 1999, 2001). In the first stage, F_t_ was presented to the participant for about 15 s. In the second stage, a series of two faces, the test-pairs, were presented, and the participant had to choose the face that most resembled F_t_ (i.e., the BEM). The results were analyzed according to the above reconstruction rule and finally F_t_ was reconstructed. The degree of success in the reconstruction was measured by V_t_ index, which counts the number of F_t_’s values that were correctly identified (ranging from 0 to 5).

The findings showed that V_t_ decreases very quickly with increasing n (number of values per dimension). It turned out that for *n* = 2, 3 the reconstruction of F_t_ was very high, but for *n* = 9 the reconstruction was poor; about one F_t_ value was identified (see Rakover & Cahlon, 2001, Fig. 7.1). (Note that in reality n exceeds 100 values.)

These findings were disappointing and frustrating. They were inconsistent with the Catch model, in fact, it seemed to me that the model had been falsified! So, what do we do now, should I abandon the model? I could not do that! I examined the findings, everything related to the experiment, and finally, as a result of feverish thinking, light appeared at the end of the dark tunnel.

The rescue from disappointment with the empirical results and the conflict of whether or not to abandon the model was based on the following considerations. In the Catch model, n is an essential factor for the generation of faces: S_F_=n^5^. As n increases, the number of faces increases. And as the number of faces increased, so did the number of faces that resembled F_t_ to one degree or another, and as a result, the interference with remembering F_t_ increased. This was a reasonable explanation for the disappointing findings: the similarity between faces and F_t_ interferes with F_t_ recognition. However, it was precisely at this point that a hopeful thought arose. Perhaps what the individual remembers is not only F_t_ in detail but also a group of faces that resemble it. This was an encouraging idea that removed the grayness of disappointment. It turned the factor that caused the failure of the Catch model, the similarity between faces, from a negative factor into a positive one that contributes and extends the model. That is, the extended Catch model reconstructs F_t_ and the faces that resemble it by applying the following experimental procedure.

Indeed, I quickly planned a series of experiments to test the possibility that the reconstruction of F_t_ involves different faces that resemble F_t_. Experimentally, this has been done by dividing n in each dimension into several subgroups, each containing a number of similar features (values). In this way, even if the reconstruction failed to hit the exact specific feature of F_t_, in many cases the reconstructed feature belonged to the similarity subgroup of F_t_. For example, assuming that dimension A (*n* = 9) includes three similarity subgroups: (a_1_ a_2_ a_3_), (a_4_ a_5_ a_6_) and (a_7_ a_8_ a_9_) and the facial feature of F_t_ is a_3_. Given this, even if the reconstruction does not include a_3_, but rather a_1_ or a_2_, the reconstruction will be considered successful because it is based on facial features that are very similar to the value of F_t_ (a_3_), i.e., the faces include the value a_1_ or the value a_2_.

The results of the experiments were very good (e.g., Rakover & Cahlon, 2008). To make a long story short, the idea of dividing each dimension into several similarity subgroups, opened up a new horizon that eventually led us to publish a paper reporting a new discovery: “Face Recognition by Similarity (FRBS) conjecture”. I cannot describe that paper here; as this would be an extension beyond the purpose of the current article (see Rakover & Cahlon, 2008). (In fact, the paper is very complex, both theoretically and empirically. The underlying idea is that face recognition is based on the degree of similarity between the test face and a set of facial representations in memory. Face similarity is an automatic and very fast process.) From the current methodological perspective, one may conclude that the decision to continue with the Catch model was effective and worthwhile. It turned out that the visual memory includes not only the exact F_t,_ but also a collection of faces that resemble it. This suggests that the methodological idea to open up the TCED in order to correct, extend the Catch model, was a good course of action that yields new and interesting findings.

In view of the above, it seems that the current example succeeds in illustrating the major methodological idea that every theory defines theoretically and empirically a provisory closed domain of research, in which that theory succeeds in explaining the phenomenon under study. However, as soon as the research’s findings are not consistent with the theory (as with n in the Catch model) one is suggested to open the TCED and to attempt to change the theory or its auxiliary hypotheses.

## Discussion

The main goal of the present article is to present the TCED as an important explanatory tool. It is able to explain two important problems that bothered the falsification approach. First, the *Duhem problem*. The TCED is a narrow theoretical-empirical domain of a theory. As such, it allows a researcher to test practically and effectively a limited number of auxiliary hypotheses and means and by this to find out why the prediction under investigation failed. In light of this, one can suggest ideas for further research.

Second, the problem of the *contradictory-findings disregard (CFD)*. The TCED suggests the following reasons why researchers are in no hurry to abandon a disconfirmed theory (see Fig. 1). (A) Despite the particular falsification, the theory under investigation has a number of successes in its research domain. (B) Abandoning the theory and seeing it as incorrect brings back the researcher to the starting point where he is faced with unknown land with an additional feeling of failure and disappointment. And (C) the TCED, as has been pointed out above, allows the disconfirmed theory to be re-examined and re-tested in a practical way, which may lead to new discoveries, as has happened with the Catch model.

These ideas require further clarifications of two issues: the first is related to the temporality of the closed explanatory domain, and the second is related to the fact that researchers attempt to decipher the way nature behaves (why and how), i.e., to unravel the unknown true theory (T_T_).

### Temporality

Figure 1 presents a researcher with a problem. On the one hand, the first three experimental predictions, P_1_ to P_3_, produced good results that supported T. However, on the other hand, P_4_ was disconfirmed. The prediction was far from the empirical result. What should the researcher do? Should he/she (a) abandon T because it is disconfirmed or (b) continue to examine T and empirically test it, that is, rigorously scrutinize the theoretical and empirical components of the experiment, and also conduct a new series of experiments? According to TCED, it is recommended that the researcher choose option (b) - the option I have chosen when the Catch model was disconfirmed by the experiments in which n (number of values per dimension) increased.

In fact, choosing option (b) is nothing more than opening up TCED to possible changes in the theory itself (or the axillary hypotheses). This is what has happened with the Catch model as a result of introducing similarity as a crucial variable into that model. (This can be seen as an example of how the ‘hard core’ of the model is expanded.) In view of this, one conclusion emerges, scientific knowledge is temporary and it changes over time. This is why the TCED is provisionally closed.

### The unknown true theory (T_T_)

The T_T_ can be seen as part of the implicit metaphysical assumptions about the world, such as the assumptions that the world is ordered and that humans can understand it. In the spirit of this approach, Thagard (2004, p. 364) wrote, “Most scientists talk and act as if they are trying to figure out how the world actually works, not just attempting to make accurate predictions. Moreover, the impressive technological successes of science are utterly mysterious unless the scientific theories that made them possible are at least approximately true.” Indeed, as an experimental psychologist, I feel that this passage reflects my view. Given this, one may suggest that the degree to which a proposed theory approaches the truth, the T_T_, could be estimated by the following theoretical general equation:

Distance from the true explanation (DT) = f(T – T_T_), when T represents a theory that is proposed for explaining the phenomenon under study, and f is estimated by using the appropriate theory and experiments.

If a correspondence is created between T and T_T_ in TCED, T is considered success and researchers continue to hold onto it and try to explain with it new phenomena. Given this situation, the following question arises: Can one conclude that in TCED T is approaching the true explanation, T_T_? The answer is divided into two parts.

On the one hand, as long as the system is conceived of as closed and T is success, one may suggest that T is approaching the true explanation, T_T_, simply because all T’s predictions match the empirical observations within TCED. On the other hand, if one understands that (a) this system is only temporarily closed and in fact, it may be opened up at any second to incomprehensible behavioral phenomena, and (b) T_T_ is not known, a mystery, the answer is completely different. Given this understanding, one may reach the following three reasonable conclusions.

- First, the scientific research is a never-ending process. This conclusion corresponds with Popper (1995, p.53) who wrote, “The game of science is, in principle, without end.”
- Second, we will never discover the truth, because T_T_ is unknown. Even if, by some chance, we will happen upon T that is identical to T_T_, we will have no way of knowing that the T_T_ has been discovered. All we could do is to test T repeatedly; tests that T will successfully pass. Similarly, Godfrey-Smith (2008, p. 145) claimed that: “…we can never believe, at any specific time, that we have found a theory that is *true*”.
- Third, the science of experimental psychology advances by eliminating incorrect, disconfirmed theories. It is impossible to know the extent to which T approaches the T_T_, since T_T_ is not known. Therefore, the proximity of T to T_T_ is estimated only in relation to competing alternative theories. Similarly, Godfrey-Smith (2008, p. 146) wrote, “The strategy employed by science would be, at any point, to use data to show that T_1_ is better than T_2_, where the hope is that this fallibly indicates that T_1_ is closer to the truth than T_2_.” Thus, the article suggests that according to TCED, the success of T is seen only as the better than alternative competing theories.

In view of the above, TCED can assist any researcher by drawing attention to (a) the empirical domain in which the theory under investigation can be tested, and (b) the definition of this domain primarily by BEM. Drawing attention to this will clarify to the researcher what has been done and what needs to be done by continuing the research.

## Data Availability

No datasets were generated or analysed during the current study.

## References

[CR1] Duhem, P. (1996). *Essay in the history and philosophy of science*. Indianapolis And Cambridge: Hackett Publishing Company (Translated by Ariew, A and Barker, P.).

[CR2] Festinger, L. (1957). *A theory of cognitive dissonance*. Row, Peterson.

[CR3] Feyerabend, P. K. (1975). *Against method*. New Left Books.

[CR4] Fiedler, K., Kutzner, F., & Krueger, J. I. (2012). The long way from α-error control to validity proper: problems with a short-sighted false-positive debate. *Perspectives on Psychological Science*, *7*, 661–669. 10.1177/1745691612462587 26168128

[CR5] Gigerenzer, G., Krauss, S., & Vitouch, O. (2004). The null ritual: what you always wanted to know about ignorance testing but were afraid to ask. In D. Kaplan (Ed.), *The Sage handbook of quantitative methodology for the social science* (pp. 391–408). Sage.

[CR6] Godfrey-Smith, P. (2008). Recurrent transient underdetermination and the glass half full. *Philosophical Studies*, *137*(1), 141–148.

[CR7] Hempel, C. G. (1979). Scientific rationality: Analytic vs. pragmatic perspectives. In T. G. Geraets (Ed.), *Rationality Today* (pp. 45–66). Ottawa University.

[CR8] Hepburn, B., & Andersen, H. (2021). Scientific method. In E. N. Zalta (Ed.), *The Stanford Encyclopedia of Philosophy*.

[CR9] Khun, T. (1970). *The structure of scientific revolutions* (2ed.). University of Chicago Press.

[CR10] Lakatos, I. (2014). Falsification and the methodology of scientific research programmes. In L. Patton (Ed.), *Philosophy, Science, and History* (pp. 170–196). Routledge.

[CR11] Leppink, J., O’Sullivan, P., & Winston, K. (2017). Evidence against vs. favour of null hypothesis. *Perspectives on Medical Education*, *6*, 115–118. 10.1007/s40037-017-0332-6 PMC5383568 28210972

[CR12] Maurer, D., Le Grand, R., & Mondloch, C. J. (2002). The many faces of configural processing. *Trends in Cognitive Sciences*, *6*, 255–260. 10.1016/s1364-6613(02)01903-4 12039607

[CR13] McCall, R. B., & Kagan, J. (1990). *Fundamental statistics for behavioral sciences, 5*^*ed*^. HBJ.

[CR14] McGuire, W. J. (2013). An additional future for psychological science. *Perspectives on Psychological Science*, *8*, 414–423. 10.1177/1745691613491270 26173120

[CR15] Neal, J. M., & Liebert, R. M. (1986). *Science and behavior: An introduction to methods of research* (3ed.). Prentice-Hall.

[CR16] Popper, K. R. (1995). *The logic of scientific discovery*. Routledge.

[CR17] Rakover, S. S. (1990). *Metapsychology: Missing links in behavior, mind and science*. Paragon/Solomon.

[CR18] Rakover, S. S. (2002). Featural vs. configurational information in faces: A conceptual and empirical analysis. *British Journal of Psychology*, *93*, 1–30. 10.1348/000712602162427 11839099

[CR19] Rakover, S. S. (2003). Experimental psychology and Duhem’s problem. *The Journal for the Theory of Social Behaviour*, *33*, 45–66.

[CR20] Rakover, S. S. (2013). Explaining the face-inversion effect: the face-scheme incompatibility (FSI) model. *Psychological Bulletin and Review*, *20*, 665–692. 10.3758/s13423-013-0388-1 23381811

[CR21] Rakover, S. S. (2018). *How to explain behavior: A critical review and a new approach*. Lexington Books.

[CR22] Rakover, S. S., & Cahlon, B. (1989). To catch a thief with a recognition model: The model and some empirical results. *Cognitive Psychology*, *21*, 423–468. 10.1016/0010-0285(89)90015-7 2791507

[CR23] Rakover, S. S., & Cahlon, B. (1999). The Catch Model: A solution to the problem of saliency in facial features. *Spatial Vision*, *12*, 73–81. 10.1163/156856899x00049 10195389

[CR24] Rakover, S. S., & Cahlon, B. (2001). *Face recognition: Cognitive and computational processes*. Benjamins.

[CR25] Rakover, S. S., & Cahlon, B. (2008). A face recognition by similarity (FRBS) conjecture. *Perception & Psychophysics*, *70*, 969–982. 10.3758/pp.70.6.982 18717384

[CR26] Siegel, H. (1985). What is the question concerning the rationality of science? *Philosophy of Science*, *52*, 517–537.

[CR27] Tanaka, J. W., & Farah, M. E. (1993). Parts and their configuration in face recognition. *Quarterrly Journal of Experimental Psychology*, *46A*, 225–245. 10.1080/14640749308401045 8316637

[CR28] Thagard, P. (2004). Rationality and science. In A. R. Mele, & P. Rawling (Eds.), *The Oxford handbook of rationality* (pp. 363–379). Oxford University Press.

[CR29] Wason, P. C. (1960). On the failure to eliminate hypotheses in a conceptual task. *The Quarterly Journal of Experimental Psychology*, *12*, 129–140.

[CR30] Wilkinson, N. (2013). Testing the null hypothesis: The forgotten legacy of Karl Popper? *Journal of Sports Sciences*, *31*, 919–920. 10.1080/02640414.2012.753636 23249368

[CR31] Yin, R. K. (1969). Looking at upside-down faces. *Journal of Experimental Psychology*, *81*, 141–145.

